# Investigation of risk factors for introduction of highly pathogenic avian influenza H5N1 virus onto table egg farms in the United States, 2022: a case–control study

**DOI:** 10.3389/fvets.2023.1229008

**Published:** 2023-07-25

**Authors:** Alice L. Green, Matthew Branan, Victoria L. Fields, Kelly Patyk, Stephanie K. Kolar, Andrea Beam, Katherine Marshall, Rachel McGuigan, Matthew Vuolo, Alexis Freifeld, Mia Kim Torchetti, Kristina Lantz, Amy H. Delgado

**Affiliations:** ^1^Center for Epidemiology and Animal Health, Animal and Plant Health Inspection Service, United States Department of Agriculture, Fort Collins, CO, United States; ^2^National Veterinary Services Laboratories, Animal and Plant Health Inspection Service, United States Department of Agriculture, Ames, IA, United States

**Keywords:** avian influenza, table egg, layers, H5N1, highly pathogenic avian influenza, case–control, biosecurity

## Abstract

**Introduction:**

The 2022–2023 highly pathogenic avian influenza (HPAI) H5N1 outbreak in the United States (U.S.) is the most geographically extensive and costly animal health event in U.S. history. In 2022 alone, over 57 million commercial and backyard poultry in 47 U.S. states were affected. Over 75% of affected poultry were part of the commercial table egg production sector.

**Methods:**

We conducted a case–control study to identify potential risk factors for introduction of HPAI virus onto commercial table egg operations. Univariate and multivariable analyses were conducted to compare farm characteristics, management, and biosecurity factors on case and control farms.

**Results:**

Factors associated with increased risk of infection included being in an existing control zone, sightings of wild waterfowl, mowing or bush hogging vegetation less than 4 times a month, having an off-site method of daily mortality disposal (off-site composting or burial, rendering, or landfill), and wild bird access to feed/feed ingredients at least some of the time. Protective factors included a high level of vehicle washing for trucks and trailers entering the farm (a composite variable that included having a permanent wash station), having designated personnel assigned to specific barns, having a farm entrance gate, and requiring a change of clothing for workers entering poultry barns.

**Discussion:**

Study results improve our understanding of risk factors for HPAI infection and control measures for preventing HPAI on commercial U.S. table egg farms.

## 1. Introduction

Many species of birds are susceptible to influenza A viruses. Aquatic wild birds constitute a major reservoir of these viruses, which are classified into subtypes according to their hemagglutinin (H) and neuraminidase (N) antigens. Low pathogenicity H5 and H7 influenza viruses generally cause few clinical signs or asymptomatic infections in poultry. While the clinical signs of highly pathogenic avian influenza (HPAI) viruses may vary, severe clinical signs and high mortality rates may occur (1).

Birds infected with influenza shed virus in feces and respiratory secretions. Disease may spread through direct contact with infected birds or their secretions or through contaminated feed and water. Indirect spread through fomites, such as contaminated farm equipment, can also spread the virus. As part of the U.S. response to HPAI, a 10 km control zone is established around each infected premises with birds kept primarily for the purpose of producing poultry or poultry products offered for sale or trade. Poultry, poultry products, and other materials from within a control zone require permitting before movement. Extensive surveillance of commercial and backyard flocks is conducted prior to control area release. Economic impacts of HPAI are wide-ranging, including not only loss of birds due to death or depopulation, but also the high cost of outbreak response and market losses associated with international trade restrictions (2).

The U.S. Geological Survey’s National Wildlife Health Center conducts surveillance in wild birds to assist in early detection of high consequence pathogens such as HPAI (3). As described by Caliendo et al. (4), the first 2021 detection of Eurasian strain H5N1 HPAI H5N1 clade 2.3.4.4b in North America occurred in December in Newfoundland and Labrador, Canada, in a mixed species flock (4). At the time, it was noted that this was the first detection of H5 influenza virus of this lineage in the Americas since June 2015 in domestic birds and December 2016 in wild birds (5). The last introduction of viruses from this lineage to North America in 2014 ultimately resulted in what was, at the time, the largest animal health emergency in the history of the United States (6).

In February 2022, the first commercial poultry flock in the U.S. was affected with this Eurasian lineage H5N1 HPAI virus. By the end of 2022, over 57 million commercial and backyard poultry in 47 U.S. states were affected (7), resulting in over $659 million in federal expenditures for control efforts and indemnity payments. While approximately 70% of all affected commercial poultry farms in 2022 were turkey farms, the commercial table egg industry was also heavily affected. Over 75% of affected commercial poultry were table egg birds. Results of full genome sequencing indicate that independent wild bird introductions have been the primary mechanism of introduction of virus into operations in this outbreak (Youk S, Torchetti MK, Lantz K, Lenoch JB, Killian ML, Leyson C, et al., pending submission). In comparison, the severity of the 2014–2015 U.S. HPAI H5N2 outbreak, once it reached the Midwest, was heavily influenced by lateral transmission of virus between farms (8, 9).

Circulation of this highly infectious HPAI virus among North American wild birds calls for updated epidemiologic investigation of factors associated with spillover infection to domestic poultry. To explore these factors, as well as how they may differ from risk factors noted in 2015, a case-control study for H5N1 HPAI was conducted among commercial table egg layer, pullet, and breeder bird farms in Delaware, Iowa, Maryland, Minnesota, Nebraska, Ohio, Pennsylvania, and Utah. This study was conducted by the United States Department of Agriculture (USDA) Animal and Plant Health Inspection Service (APHIS) in collaboration with State partners, academia, and national poultry organizations. Goals of this study included identifying risk factors for HPAI on commercial table egg farms, identifying biosecurity challenges on commercial table egg farms, and providing data to assist in refining biosecurity recommendations to support prevention of HPAI. This information will improve understanding of risk factors associated with HPAI on table egg farms in the United States to support science-based guidance on farm-level preventative measures.

Unprecedented transmission of this H5N1 clade 2.3.4.4b among wild bird populations results in ongoing high risk for domestic poultry (10) and significant economic impacts for affected producers. The USDA’s National Veterinary Services Laboratories perform whole genome sequencing (WGS) of the influenza virus for all confirmed positive operations and conduct analyses to help determine whether the sequence (s) are consistent with independent wild bird-origin introduction or represent the potential for lateral spread while considering all available epidemiologic data. The subset of operations likely to be infected by wild bird introduction was further examined in relation to selected farm characteristics and biosecurity-related management practices to determine possible associations with wild bird-related spillover risks for HPAI.

In addition to this commercial table egg farm study, there was a similar but independent study of the risk factors for HPAI affecting turkey farms, also conducted by USDA–APHIS. Despite similar overall objectives, the study design and target population were sufficiently different to warrant a separate report. This publication relates to the table egg layer investigation, while the turkey farm investigation is reported separately.

## 2. Materials and methods

### 2.1. Case definition and laboratory testing

Samples from poultry farms were screened for influenza A, and H5/H7 subtypes by reverse transcript real-time polymerase chain reaction (RT-PCR) by members of the National Animal Health Laboratory Network (NAHLN). Samples testing non-negative by influenza A virus (IAV) PCR were sent to the National Veterinary Services Laboratories (NVSL, Ames, Iowa, United States) for confirmation. Testing at NVSL included an H5 clade 2.3.4.4 pathotyping assay and an assay targeting N1 for neuraminidase subtyping. Whole genome sequencing was conducted directly from the samples. Influenza A viruses were sequenced directly from samples as previously described (11); RAxML was used to generate phylogenetic trees, and tables of single nucleotide polymorphisms (SNPs) were created using the vSNP pipeline.^1^

For the purposes of this study, a case farm was defined as any U.S. table egg layer, pullet, or breeder premises in Delaware, Iowa, Maryland, Minnesota, Nebraska, Ohio, Pennsylvania, or Utah from which samples were confirmed positive from February through September 2022. Control farms were defined as farms from the same states that did not have HPAI during the study period.

### 2.2. Questionnaire design and data collection

By September 30, 2022, more than 29 million table egg layers, pullets, and breeder birds in the participating 8 states had been lost from infection or depopulation (7). A case–control study was designed to examine risk factors associated with HPAI infection on U.S. commercial table egg farms. For the current study, the questionnaire from Garber et al. (12) was updated and condensed based on academic, field, and industry subject matter expertise. The 26-page questionnaire (included in Supplementary material) covers farm characteristics, wild birds and wildlife, biosecurity, personnel, visitors, vehicles, equipment, and management practices for the 14 days prior to detection of clinical signs or increased mortality on case farms, and for a comparable 14-day reference period on control farms. In rare situations where clinical signs or an increase in mortality were not noted, farmers were asked to provide the date that the farm was positive for HPAI based on diagnostic test results. This situation was most common when farms were located within a control or surveillance zone.

Eligible case farms included those commercial table egg layer, pullet, and breeder farms in Delaware, Iowa, Maryland, Minnesota, Nebraska, Ohio, Pennsylvania, or Utah with confirmed infection from February 22 through September 30, 2022. A total of 22 farms met the inclusion criteria. While confirmed infections also occurred in Colorado, South Dakota, and Wisconsin, these were not included due to resource constraints or lack of eligible control premises. Eligible control farms were any commercial table egg layer, pullet, or breeder farms selected from the same states as case farms, using the USDA–APHIS Veterinary Services Emergency Management Response System. Randomized lists of 10 potential controls per case were shared with interviewers in each participating state, with a goal of enrolling up to two control farms per case farm. Potential controls were contacted by interviewers via phone or email to confirm eligibility and interest in participating. To be considered eligible, control farms needed to have 50,000 or more birds, as well as birds on site for a minimum two-week window of risk within the state-specific high-risk timeframe. High-risk timeframes were determined according to reported onset of clinical signs for confirmed infections within the states. Interviewers were asked to match risk windows for cases and controls as closely as possible.

Questionnaires were administered by Federal or State veterinary medical officers via telephone interviews. Fillable pdf forms were uploaded to a secure APHIS location. Interviewers in each state only had access to their state’s data. All data were treated as confidential business information; due to this requirement, results are shared in aggregate only. Producer participation was voluntary. Questionnaire administration took place between September 26 and December 28, 2022.

### 2.3. Data entry and management

Survey data were entered into a SAS dataset using SAS version 9.4 (SAS Institute Inc., Cary, NC). Survey responses were validated by USDA–APHIS staff prior to analysis. Validation included reviewing survey responses for consistency and logical issues such as the proper treatment of skip patterns (e.g., if a respondent reported not having a certain type of visitor on the operation but then also reported a count of more than zero visits made to the operation by that visitor type), checking for invalid responses (e.g., a response of “0” for a 1/3 response variable), reclassification of other specify responses (e.g., if the respondent chose another type of road surface but wrote in a surface type that was consistent with the listed road surface types), and other conditional logic checks. Random, single imputation was used for variables included in multiple regression modeling for which there was item nonresponse, but a valid response could not be deduced using the validation steps outlined above (13, 14).

Validation and univariable analyses were performed using SAS version 9.4. Multiple regression modeling was performed using R version 4.1.1 (15), implemented within R Studio version 1.4.1717 (16). The R packages used included AICcmodavg (17), blorr (18), BMA (19), car (20), caret (21), haven (22), Hmisc (23), leaps (24), lme4 (25), and tidyverse (26). Exact multiple logistic regression model estimates were generated using PROC LOGISTIC in SAS version 9.4.

### 2.4. Univariable analyses

Univariable analyses were performed to identify variables potentially associated with the presence of HPAI, and a Fisher’s exact test was used for categorical variables to assess the association of each variable with HPAI infection. Numeric variables were broken into quartiles for assessment. Variables with Fisher’s exact test *p* ≤ 0.20 that were also biologically plausible for risk of HPAI infection and had at least 5 responses per level were considered for entry into candidate multivariable models.

The subset of farms that had WGS results consistent with wild bird introduction was further examined in relation to selected farm and biosecurity-related factors to determine which of these may be specifically associated with wild bird-related spillover risk for HPAI.

### 2.5. Multivariable analyses

#### 2.5.1. Multicollinearity and confounding variables

From the pool of screened predictor variables, variance inflation factors (VIFs) (27) were computed. Variables with VIFs exceeding 3 indicated further investigation was needed. All of the predictor variables considered were binary, so the ordinary VIF was used rather than the generalized VIF (28). Groupings of variables with high similarity were identified using hierarchical clustering on a similarity matrix, calculated using proportions of observations that were positive for each pair of binary predictor variables, using complete linkage clustering (29).

Confounding and effect measure modification were assessed using three statistical methods combined with subject-matter expertise regarding likely causal relationships between predictor variables and HPAI presence (30, 31). First, the relationship between the variable of interest and the confounding variable and the relationship between the confounding variable and HPAI presence were both assessed using logistic regression modeling and assessing statistical significance at the 0.10 significance level. Secondly, the relative change in the estimated odds ratio associated with the predictor variable of interest in a multiple logistic regression model prior to and after inclusion of the potential confounding variable was assessed, with changes of more than 10 percent being indicative of potential confounding. Lastly, biological, and epidemiological plausibility was used to determine whether the potential confounding variable was in the causal pathway between the variable of interest and HPAI presence. If a potential confounding variable passed the statistical checks and was found not to be in the causal pathway between the predictor of interest and HPAI presence, then that variable would be adjusted for in the multiple logistic regression model. Potential confounding relationships were tested to identify whether they were indicative of confounding, effect measure modification, or both (31).

#### 2.5.2. Leave-one-out cross-validation and AICc

Leave-one-out cross-validation [LOOCV] (32) was used to rank models according to their ability to classify case and control farms. In this study, because there were approximately equal numbers of case and control farms in the dataset, the overall goodness of the multiple logistic regression model predictions was taken as the accuracy of the predictions made using the models.

The values of the accuracy of the models ranked using LOOCV were relatively coarse due to sample size. Therefore, a second model goodness criterion was used to order models within a given accuracy. The second model goodness criterion was AICc, which is a variant of Akaike’s information criterion (33) with an adjustment for small sample sizes (34). Smaller values of AICc indicate models that are expected to approximate the underlying process more closely than models with higher values of AICc.

#### 2.5.3. Multiple logistic regression modeling

During model selection, multiple logistic regression models were fit using iteratively reweighted least squares using the glm function in R, specifying the response as the indicator for whether a farm was a case (response value of 1) or a control (response value of 0) as a function of one or more predictor variables. For the final model-based estimates, an exact multiple logistic regression model wasfit using the EXACT statement in the LOGISTIC procedure within SAS, which gives conditional maximum likelihood estimates of odds ratios and their confidence intervals (35), which use permutation theory and are appropriate to use in situations in which there are small sample sizes.

Logistic regression model fit was assessed using deviance residual diagnostic plots (27) to check for influence, leverage, and overall model fit. Statistics used to describe relative model goodness included the LOOCV accuracy and AICc. McFadden’s pseudo–$R^{2}$ and McFadden’s adjusted pseudo-$R^{2}$ were used to assess explained variability of the model (36).

The primary inferential statistics derived included estimated odds ratios (OR), indicating the multiplicative increase in the odds of a farm being a case farm associated with a given predictor variable, given all other predictor variables remained constant. In addition, estimated 95% confidence intervals for the odds ratios were used to communicate uncertainty in the point estimates, and Type III F-test (37) value of ps were used to assess the statistical significance of model effects.

#### 2.5.4. Bayesian model averaging

In addition to investigating potentially important predictor variables for HPAI presence using the above methods to select a single model from which to make inference, Bayesian model averaging was used to further investigate the effects of the predictor variables in the pool under consideration. Bayesian model averaging (BMA) is a statistical method that attempts to account for the uncertainty induced by the model selection problem by taking information from a broad group of models rather than from a single model (38). BMA has been shown to improve predictive ability over single-model selection methods.

Bayesian model averaged estimates of the posterior probabilities that the predictor variable effects were non-zero, odds ratios, and approximate 95% confidence intervals for the odds ratios were produced using the BMA package in R. These estimates were used to estimate the effect of each predictor variable on HPAI presence in a multiple logistic regression model setting, accounting for the spread of effect sizes those variables take across a broad range of possible models.

## 3. Results

Eighteen of 22 (81.8%) commercial table egg farms affected by HPAI in the 8 participating states agreed to participate in the study. An estimated 20% of potential control producers met eligibility criteria and agreed to participate. Onset of clinical signs for affected flocks ranged from March 3 to August 31, 2022. To maintain confidentiality of participating producers, cases and controls were reported by region; states in the Eastern region included Maryland, Delaware, Ohio, and Pennsylvania. States in the Midwest/Western region included Iowa, Minnesota, Nebraska, and Utah. Samples from this study were collected between March 2022 and September 2022. There were 18 case farms; 11 farms in the Eastern region (61.1% of case farms) and 7 farms in the Midwest/Western region (38.9% of case farms). There were 22 control farms, with 15 of the control farms in the Eastern region (68.2% of control farms) and 7 of the control farms (31.8% of control farms) in the Midwest/Western region. All premises were tested by PCR and confirmed with H5N1 clade 2.3.4.4b at NVSL. Based on the analysis of full genome sequences and in consideration of available epidemiologic data, each layer case farm that participated in the case–control study was categorized by likely route of introduction of virus: introductions consistent with independent wild bird-origin were identified for 61% of case farms (*n* = 11), whereas potential lateral spread or common source exposure was found for 39% of case farms (*n* = 7). The case–control analysis included 18 case farms and 22 control farms. Median flock size for case farms was 900,000 (range: 72,000–5,000,000). Median flock size for control farms was 480,000 (range: 77,000–2,900,000). Of the case farms, 83% (*n* = 15) had table egg layers, and 22% (*n* = 4) had pullets or breeders. Of the control farms, 91% (*n* = 20) had table egg layers, and 14% (*n* = 3) had pullets or breeders (Please note that there was some overlap between categories). During the study period, a total of 137 control zones were active in the 8 states that took part in the study.

### 3.1. Univariable analyses

Selected results of the farm-level univariable analysis are shown in Tables 1–3. These results do not include imputed values. A complete list of univariable results is available in the Supplementary material.

**Table 1:** Univariable analyses of factors considered for entry into farm-level multivariable models.

Characteristic	Number of case farms (percent)	Number of control farms (percent)	Univariable *p*-value (Fisher’s exact)	Odds ratio (95% confidence interval)
In an existing control zone	8 (44.4)	2 (9.1)	0.02	8.0 (1.4, 44.9)
Flock size was large (≥500,000 birds)	11 (61.1)	11 (50.0)	0.54	1.6 (0.4, 5.6)
Closest field within 320 meters (350 yards) of farm tilled previous fall	2 (11.1)	9 (42.9)	0.04	0.2 (0.0, 0.9)
Wild waterfowl or shorebirds seen in closest field during reference period	8 (44.4)	2 (9.5)	0.03	7.6 (1.3, 42.8)
Farm entrance gated	4 (22.2)	14 (63.6)	0.01	0.2 (0.0, 0.7)
Vegetation mowed/bush hogged less than 4 times a month	11 (64.7)	9 (40.9)	0.20	2.6 (0.7, 9.8)
Lower level of vehicle washing (combination variable)	16 (88.9)	15 (68.2)	0.15	3.7 (0.7, 20.9)
Vehicle tires washed	11 (61.1)	18 (81.8)	0.17	0.3 (0.1, 1.5)
Feed trucks washed	9 (50.0)	18 (81.8)	0.05	0.2 (0.1, 0.9)
Egg trucks washed	9 (50.0)	16 (76.2)	0.11	0.3 (0.1, 1.2)
Company trucks/trailers either not shared or shared and always disinfected during reference period	12 (66.7)	20 (90.9)	0.11	0.2 (0.0, 1.2)
Permanent vehicle washing station	3 (16.7)	9 (40.9)	0.17	0.3 (0.1, 1.3)
Any vehicles either not shared or shared and always disinfected during reference period (combination variable)	7 (38.9)	6 (27.3)	0.51	1.7 (0.4, 6.4)

Number and percentage of case farms and number and percentage of control farms by farm, wild bird, and vehicle-related characteristics.

**Table 2:** Univariable analyses (*p* ≤ 0.20) of factors considered for entry into farm-level multivariable models.

Characteristic	Number of case farms (percent)	Number of control farms (percent)	Univariable *p*-value (Fisher’s exact)	Odds ratio (95% confidence interval)
Change of clothing always required for workers entering poultry barns	12 (66.7)	20 (90.9)	0.11	0.2 (0.0, 1.2)
Different personnel for different barns (dedicated barn personnel)	**	5 (22.7)	0.20	0.2 (0.0, 1.9)
Severity of rodents low, moderate, or high (vs. not a problem)	13 (72.2)	10 (45.5)	0.12	3.1 (0.8, 11.8)
Wild birds able to access feed/feed ingredients at least sometimes	9 (50.0)	6 (27.3)	0.19	2.7 (0.7, 10.0)
Wild animals able to access feed/feed ingredients at least sometimes	6 (33.3)	2 (9.1)	0.11	5.0 (0.9, 28.9)
Incineration as a method of daily mortality disposal	**	6 (27.3)	0.10	0.2 (0.0, 1.5)
Off-site method of daily mortality disposal (off-site composting or burial, rendering, or landfill)	9 (50.0)	6 (27.3)	0.19	2.7 (0.7, 10.0)

Number and percentage of case farms and number and percentage of control farms by premises feed, rodent-related, worker-related, and mortality disposal management practices.

**Too few to report.

**Table 3:** Univariable analyses of selected factors analyzed for the subset of farms with independent wild bird introduction of HPAI.

Characteristic	Number of case farms (percent)	Number of control farms (percent)	Univariable *p*-value (Fisher’s exact)	Odds ratio (95% confidence interval)
In an existing control zone	3 (27.3)	2 (10.0)	0.32	3.4 (0.5, 24.3)
Structural windbreak present (e.g., hill, natural break)	0 (0.0)	6 (30.0)	0.07	*
Wastewater lagoon visible or within 320 meters (350 yards) of farm	8 (72.7)	9 (45.0)	0.26	3.3 (0.7, 16.0)
Drainage ditch visible or within 320 meters (350 yards) of farm	7 (63.6)	7 (35.0)	0.15	3.3 (0.7, 15.1)
Wild waterfowl or shorebirds seen in closest field during reference period	4 (36.4)	1 (5.3)	0.05	10.3 (1.0, 108.8)
Low, moderate, or high rodent problem vs. rodents not a problem	8 (72.7)	8 (40.0)	0.14	4.0 (0.8, 19.8)
Wild birds able to access feed/feed ingredients at least sometimes	8 (72.7)	6 (30.0)	0.03	6.2 (1.2, 31.9)
Clean up feed spills immediately	5 (50.0)	16 (80.0)	0.12	0.3 (0.1, 1.4)

Number and percentage of case farms and number and percentage of control farms by premises characteristics, biosecurity, and feed-related management practices.

*OR not shown due to zero cell value for case farms.

*Farm characteristics* (Table 1). During the 14-day reference period, more case farms were located within an existing control zone compared to control farms (44% vs. 9%, OR = 8.0, *p =* 0.02). Fewer case farms were within 320 meters (350 yards) of a field that had been tilled the previous fall (11% vs. 43%, OR = 0.2, *p =* 0.04).

*Wild bird/wild animal characteristics* (Table 1). Wild waterfowl or shorebirds were seen in the closest field during the 14-day reference period on 44% of case farms compared to 10% of control farms (OR = 7.6, *p =* 0.03).

*Vehicle-related characteristics* (Table 1). Using wash stations to wash vehicle tires was more commonly reported on control farms (82% vs. 61% of case farms, OR = 0.3, *p =* 0.17). Using wash stations for feed trucks was more commonly reported on control farms (82% vs. 50% of case farms, OR = 0.2, *p =* 0.05). Using wash stations for egg trucks was more commonly reported on control farms (76% vs. 50% of case farms, OR = 0.3, *p =* 0.11). Ninety-one percent of control operations either did not share or shared and always disinfected company trucks and trailers that might be used by another farm, as compared with 67% of case operations (OR = 0.2, *p =* 0.11). Permanent vehicle wash stations were more commonly reported by control farms (41% vs. 17% of case farms, OR = 0.3, *p =* 0.17).

*Biosecurity characteristics* (Tables 1, 2). Having a gated farm entrance was more commonly reported on control farms (64% vs. 22% of case farms, OR = 0.2, *p =* 0.01). Farm mowing or bush hogging less than 4 times a month was more commonly reported on case farms (65% vs. 41% of control farms, OR = 2.6, *p =* 0.20). Incineration was more commonly reported as a method of daily mortality disposal on control farms (OR = 0.2, *p* = 0.10, Table 2).

*Worker and visitor-related practices* (Table 2), *not all data shown.* In general, use of occasional workers was uncommon. Most farms always required the use of a clean/dirty line for workers entering barns (83% of case farms vs. 91% of control farms, *p =* 0.64). Always requiring a washable change of clothing for workers entering barns was more commonly reported by control farms (91% vs. 67% of case farms, OR = 0.20, *p =* 0.11). Nearly all farms always required a change of shoes or use of shoe covers for workers entering barns. Overall, visitors to farms were not common. High percentages of both case and control farms required visitors not to visit multiple farms in the same day (92% of case farms and 84% of control farms, *p =* 1.00). Workers being assigned to specific barns (dedicated barn personnel) was more commonly reported on control farms (OR = 0.2, *p* = 0.20).

*Rodent management and wildlife feed access* (Table 2). Having at least some problem with rodents was more commonly reported on case farms (72% vs. 46% of control farms, OR = 3.1, *p =* 0.12). Having wild bird access to feed or feed ingredients at least some of the time was more commonly reported on case farms (50% vs. 27% of control farms, OR = 2.7, *p =* 0.19). Having wild animal access to feed or feed ingredients at least some of the time was more commonly reported on case farms (33% vs. 9% of control farms, OR = 5.0, *p =* 0.11).

*Other variables of interest, not statistically significant.* Among case farms, 33% reported use of a renderer as a general practice, while this mortality disposal method was reported by 14% of control farms as a general practice (*p =* 0.25). The practice of cleaning up feed spills immediately was reported by 67% of case farms and 82% of control farms (*p =* 0.30). Half of case farms and half of control farms reported having hard top/asphalt roads as the road surface on the farms that vehicles coming onto the operation drive on.

*Variables analyzed for independent wild bird introduction of HPAI* (Table 3). For the univariate analysis of case farms where HPAI was independently introduced by wild birds, being located within a control zone was not significant (27% of case farms vs. 10% of control farms, OR = 3.4, *p* = 0.32). Having a structural windbreak such as a hill or other natural break present was more common for control farms (30% vs. 0% of case farms), (OR not calculated due to zero cell, *p* = 0.07). Though not statistically significant, being within 320 meters (350 yards) of a wastewater lagoon was more common among case farms (73% vs. 45% of control farms, OR = 3.3, *p* = 0.26). Having a drainage ditch visible or within 320 meters (350 yards) of the farm was more common among case farms (64% vs. 35% of control farms, OR = 3.3, *p* = 0.15). Seeing wild waterfowl or shorebirds in the field closest to the farm during the reference period was more common among case farms (36% vs. 5% of control farms, OR = 10.3, *p* = 0.05). Having at least some problem with rodents was more common among case farms (73% vs. 40% of control farms, OR = 4.0, *p* = 0.14). Wild bird access to feed or feed ingredients at least some of the time was more common among case farms (73% vs. 30% of control farms, OR = 6.2, *p* = 0.03). Cleaning up feed spills immediately was more common among control farms (80% vs. 50% of case farms, OR = 0.3, *p* = 0.12).

### 3.2. Multivariable analyses

#### 3.2.1. Variable selection

Variables that had observed cell sizes of 5 or greater and a Fisher’s exact test *p* ≤ 0.20 included the following 19 variables.

- The farm was in an existing control zone on the reference date,
- The closest crop field was tilled last fall,
- Presence of any wild waterfowl or shorebirds in the closest crop field during the 14-day reference period,
- Egg trucks moving eggs off the farm generally came near the barns,
- Other business visitors (e.g., meter reader, repairman) generally came near the barns,
- The farm had a gated entrance,
- Frequency of mowing of vegetation on the premises,
- Tires were washed for vehicles on the farm during the 14-day reference period,
- Feed vehicles were washed during the 14-day reference period,
- Egg trucks were washed during the 14-day reference period,
- The vehicle wash station was a permanent station rather than recently put in place,
- There was any rodent problem during the 14-day reference period,
- Wild birds had access to feed or feed ingredients during the 14-day reference period,
- Wild animals (such as raccoons, opossums, coyotes, or foxes) had access to feed or feed ingredients during the 14-day reference period,
- Different personnel were always required for different barns (workers assigned to specific barns) during the 14-day reference period.
- Workers were always required to change clothes before entering barns (washable clothes, not disposable), and
- The farm had a high level of vehicle washing during the 14-day reference period (equal to 1 if the farm had a vehicle wash station, washed tires of vehicles, washed worker, feed, and egg trucks, and had a permanent wash station rather than one that was recently put in place due to heightened biosecurity measures surrounding the HPAI outbreak, and equal to 0 otherwise),
- Incineration was used to dispose of dead birds (daily mortality), and
- The farm disposed of dead birds (daily mortality) off-site (equal to 1 if the farm composted off-site, buried off-site, used a renderer, or a landfill and was equal to 0 otherwise).

Two additional variables that passed the univariable screening and had sufficient cell sizes included whether a farm had egg trucks moving eggs off the farm come near the barns or whether other business visitors (e.g., a meter reader, repairman) come near the barns. These variables were not considered for multiple regression modeling because their effects appeared to be counter to an epidemiological explanation and deserve further investigation which could not be thoroughly performed due to the lack of variability in the study responses For example, all case farms (*n* = 2) and all control farms (*n* = 11) that allowed egg trucks near the barns reported washing egg trucks during the reference period. Of farms that allowed company personnel vehicles to come near the barns, 71.4% (*n* = 5) of case farms and 80.0% (*n* = 8) of control farms reported washing worker vehicles during the reference period.

One variable that did not pass the univariable screening but was included in multiple regression modeling was an indicator variable for flock size, measured as the number of birds on the farm on the reference date, where farms with fewer than 500,000 birds were considered small, and those with 500,000 birds or more were considered large. The cutoff of 500,000 birds was just below the median flock size. This variable was included as it is related to several of the poultry and farm management factors listed above and was involved in a confounding relationship with at least one of them.

#### 3.2.2. Multicollinearity and confounding variables

Of the variables that met the cell size requirements and passed univariable screening, the four individual vehicle washing indicator variables (items h-k in the list above) were omitted in favor of the high level of washing combination variable (item q) due to the resulting high VIF values. In addition, wild animal access to feed (item n) was omitted in favor of the variable measuring wild bird access to feed (item m) due to high VIF values. The closest crop field being tilled the previous fall (item b) appeared to be multicollinear with the presence of any wild waterfowl in the closest crop field (item c), and so the former was omitted in favor of the latter. Finally, incineration as a method of dead bird disposal (item r) was omitted in favor of the combination variable assessing any off-site method of disposal (item s) due to high VIF values. This left 12 variables that were used in multiple regression modeling. Those variables are described in Table 4.

**Table 4:** Predictor variables included in multiple regression modeling, including name, description, and calculation of the variable.

Name	Description	Calculation
Flock size	Number of birds on the farm on the reference date. Categorized as farms having 500,000 birds or more versus farms having fewer than 500,000 birds.	if sum(e204, e210) > = 500,000 then 1 else 0
Control zone	Farm was in an existing control zone on the reference date versus the farm was not in an existing control zone on the reference date.	if e205 = 1 then 1 else 0
Waterfowl presence	Any waterfowl (e.g., ducks, geese) or shorebirds seen in the closest crop field during the reference period versus no waterfowl or shorebirds seen in the closest crop field.	if e354 in (2–4) then 1 else 0
No farm entrance gate	Farm had a gated entrance versus the farm did not have a gated entrance.	if e417 = 3 then 1 else 0
Wild bird access to feed	Wild birds had any access to poultry feed or feed ingredients during the reference period versus wild birds had no access to poultry feed or feed ingredients.	if e449 in (1–3) then 1 else 0
Off-site disposal	Farm disposed of dead birds (daily mortality) using off-site methods (composting off-site, burial off-site, rendering, or landfill) versus the farm used other disposal methods.	if e1102a = 3 or e1103a = 3 or e1105 = 1 or e1106 = 1 then 1 else 0
No dedicated barn personnel	Different personnel were assigned to specific barns during the reference period versus moving between barns.	if e606 = 1 then 1 else 0
At least some rodent problems	Farm had any rodent problem (low, moderate, or high severity) during the reference period versus the farm had no rodent problem.	if e445 in (1–3) then 1 else 0
Change of clothing not always required for workers	A change of (washable) clothing was always required for workers entering barns during the reference period versus a change of clothing was not always required.	if e608 = 1 then 1 else 0
Sharing company trucks/trailers	Farm shared and either sometimes or never cleaned and disinfected company trucks/trailers (e.g., pickup truck, trailer with supplies, supervisor truck, or similar) during the reference period versus the farm did not share company trucks/trailers or shared and always cleaned and disinfected them.	if e801 = 1 and e801a in (2, 3) then 1 else 0
Mowing less than 4 times/month	Farm mowed or bush hogged vegetation on the premises (when vegetation is present, e.g., spring and summer) 3 or fewer times per month versus 4 or more times per month.	if e420 < = 3 then 1 else 0
Lower level of vehicle washing	Farm had a vehicle wash/spray station during the reference period and washed tires of vehicles, washed worker, feed, and egg vehicles, and the wash station was permanent (e.g., in use prior to the HPAI incident) rather than a station that was recently put into use as a response to heightened biosecurity concerns versus the farm did not practice at least one of these.	if e421 = 1 and e423 = 1 and e426 = 1 and e427 = 1 and e428 = 1 and e431 = 1 then 0 else 1

All variables are binary and, as shown, take the value 1 for the category associated with a higher risk of HPAI. The calculations refer to the item codes from the questionnaire for each variable (e.g., e205 is the item code that holds a 1 or a 3, indicating whether the farm was in a control zone on the reference date from item 2.d on the questionnaire).

There were five confounding relationships identified using the statistical screening measures and in which the confounding effect was not believed to lie in the causal pathway between the predictor variable of interest and HPAI presence. Those included: waterfowl presence confounding flock size, control zone and the presence of a farm entrance gate were confounded, control zone confounding the sharing of company trucks/trailers, and the presence of a farm entrance gate confounded the presence of a high level of vehicle washing.

The sample size was not sufficient to adequately assess whether these relationships were confounding, effect measure modification, or both. However, for the relationships between waterfowl presence and flock size, control zone and the sharing of company trucks/trailers, and the presence of a farm entrance gate and the presence of a high level of vehicle washing, the evidence leaned more in favor of confounding relationships (both conditional ORs close to one another and both below the crude OR). The relationship between control zone and the presence of a farm entrance gate suggested there was effect measure modification present, with farms in a control zone tending to have higher ORs of having HPAI present if they did not have a farm entrance gate, while farms outside of the control zone tended to have lower (though still greater than 1.0) ORs of having HPAI present if they did not have a farm entrance gate. These effects were controlled for in multiple logistic regression modeling, but interaction effects were not included due to inadequate sample size.

#### 3.2.3. Leave-one-out cross-validation and AICc

All possible models with no interaction terms were created using the remaining 12 variables for a total of 4,095 models (the intercept-only model was not considered). The top 15 models ranked by LOOCV accuracy and AICc are depicted in Table 5. The variables included in each of the top 15 models and the count of which models include them are also indicated. McFadden’s unadjusted and adjusted pseudo-$R^{2}$ values are depicted for each model as well.

**Table 5:** Predictor variables included (indicated by a black box) in the top 15 models for HPAI presence, ranked by leave-one-out cross validation (LOOCV) accuracy and AICc.

Model	LOOCV Accuracy	AICc	$R^{2}$	$R_{adj}^{2}$	Control zone	Waterfowl presence	No farm entrance gate	Flock size	No specific barn personnel	Wild bird access to feed	At least some rodent problems	Lower level of vehicle washing	Change of clothing not always required	Sharing company trucks/ trailers	Mowing less than 4 times/mo.	Off-site disposal
1	77.5	48.8	0.38	0.16	⬛	⬛	⬛	⬛	⬛							
2	77.5	51.7	0.38	0.12	⬛	⬛	⬛	⬛	⬛			⬛				
3	75.0	47.5	0.35	0.17	⬛	⬛	⬛			⬛						
4	75.0	48.0	0.45	0.19	⬛	⬛	⬛	⬛		⬛			⬛			
5	75.0	48.7	0.33	0.15	⬛	⬛	⬛								⬛	
6	75.0	50.3	0.35	0.13	⬛	⬛	⬛			⬛		⬛				
7	75.0	50.5	0.40	0.15	⬛	⬛	⬛		⬛	⬛	⬛					
8	75.0	50.7	0.40	0.14	⬛	⬛	⬛	⬛	⬛		⬛					
9	75.0	51.0	0.45	0.16	⬛	⬛	⬛	⬛	⬛	⬛	⬛					
10	75.0	51.3	0.33	0.11	⬛	⬛	⬛						⬛		⬛	
11	75.0	51.7	0.38	0.12	⬛	⬛	⬛	⬛	⬛					⬛		
12	75.0	53.8	0.40	0.11	⬛	⬛	⬛	⬛	⬛		⬛	⬛				
13	75.0	54.8	0.38	0.09	⬛	⬛	⬛	⬛	⬛			⬛		⬛		
14	72.5	48.4	0.44	0.19	⬛	⬛	⬛	⬛		⬛	⬛					
15	72.5	48.5	0.38	0.16	⬛	⬛	⬛		⬛	⬛						
Count					15	15	15	9	9	7	5	4	2	2	2	0

Variables included in each model are depicted and a count of models in the top 15 in which each predictor variable is depicted in the last row. McFadden’s pseudo-$R^{2}$ and McFadden’s adjusted coefficient of determination, pseudo-$R_{adj}^{2}$ are included for each model.

Control zone, waterfowl presence, and no farm entrance gate were included in all 15 of the top models. Flock size and not always having different personnel for different barns were included in 9 of the top 15 models, and wild bird access to feed was included in 7. The remaining variables were included in 5 or fewer models, indicating lower importance regarding their ability to predict which farms were positive, given the other variables included in the models. LOOCV accuracies ranged from 0.725–0.775 in the top 15 models. This indicates that, given an exchangeable set of new table egg layer farms in the HPAI 2022 outbreak, we would expect these models to accurately predict the HPAI infection status of between 72.5 and 77.5 percent of the farms – though there is evidence that this accuracy estimate is likely biased high (32, 39). McFadden’s pseudo-$R^{2}$ values range from 0.33–0.45 for the top 15 models, with the top model having a value of 0.38. Values of McFadden’s pseudo-$R^{2}$ are typically lower than those from ordinary linear regression, where values between 0.2 and 0.4 have been cited as indicating a good amount of variability explained in the response by the given logistic regression model (36). The adjusted McFadden’s coefficient of determination, pseudo-$R_{adj}^{2}$, values ranged from 0.09 to 0.19, with the top model having a value of 0.16.

The model with the highest LOOCV accuracy and the lowest AICc is summarized in Table 6 using exact multiple logistic regression modeling to account for small sample size. None of the predictor variables were statistically significant at the 0.05 significance level, though whether the farm was in an existing control zone on the reference date (*p* = 0.09) was the most important variable. Control zone had an odds ratio of 10.3 (95% CI: 0.8–377.0), indicating that a farm that was in a control zone had an expected odds of being positive for HPAI 10.3 times greater than that for a farm that wasn’t in a control zone. Wild waterfowl or shorebird presence in the closest crop field had an odds ratio of 5.8 (95% CI: 0.7–79.4), meaning that farms that had observed wild waterfowl or shorebirds in the closest crop field during the reference period had an expected odds of being positive for HPAI about 5.8 times greater than farms that did not, though this effect wasn’t significant at the 0.05 significance level (*p* = 0.12). Not having a gate at the farm entrance had an OR of 3.8 (95% CI: 0.6–31.5), not always requiring different personnel working in different barns had an OR of 6.2 (95% CI: 0.3–427.5), and larger operations had an OR of 2.6 (95% CI: 0.3–39.5), though none of these effects were statistically significant (value of ps of 0.21, 0.34, and 0.59, respectively).

**Table 6:** Summary of the exact multiple logistic regression model fit regressing HPAI disease status of an operation on risk factors, with the greatest leave-one-out cross validation accuracy and lowest AICc value among all models under consideration.

Variable	Level	Exact conditional test *p*-value	OR Point estimate	OR 95% Confidence Interval
Intercept			0.0	(0.0, 0.5)
Flock size (number of birds on the farm on the reference date)	Large (≥500,000)	0.59	2.6	(0.3, 39.5)
	Small (<500,000)		(referent)	
Farm in an existing control zone on the reference date	Yes	0.09	10.3	(0.8, 377.0)
	No		(referent)	
Wild waterfowl or shorebirds in closest crop field during the 14-day reference period	Yes	0.12	5.8	(0.7, 79.4)
	No		(referent)	
Was there a gate to the farm entrance	Yes	0.21	(referent)	
	No		3.8	(0.6, 31.5)
Were there always different personnel working in different barns	Yes	0.34	(referent)	
	No		6.2	(0.3, 427.5)

A separate, mixed effects multiple regression model was fit using the fixed effects that were included in the LOOCV top model, plus a random intercept term for state to assess state-level farm location effects. The percentage of variance explained by state in the model was 0.8%, and so it was decided that final inference would be made from the exact multiple logistic regression model, with fixed effects only, as depicted in Table 6.

#### 3.2.4. Bayesian model averaging

**Figure 1:**
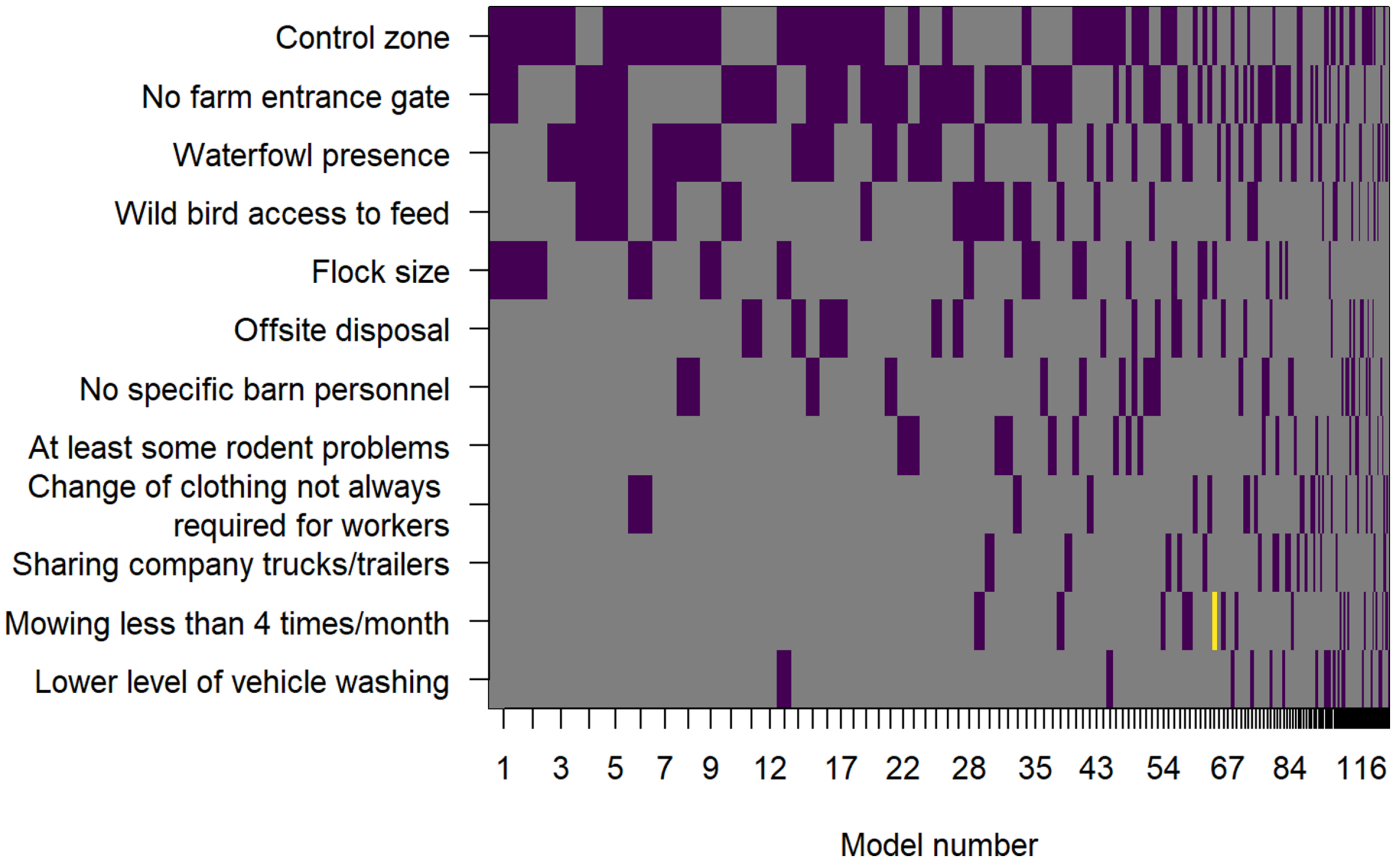
Image plot showing the variables included in the top models included in the Bayesian model averaging process. Predictor variables are on the vertical axis and models are on the horizontal axis. Each model composes a vertical stack of rectangles with width proportional to the Bayesian posterior probability of the model being selected. The rectangles are colored purple where the predictor variable was a risk factor in that given model, yellow if it was protective, and grey where that predictor variable was not included in the model.

The same 12 predictor variables that were used in the LOOCV model selection procedure were used in the Bayesian model averaging procedure. An image plot showing variable inclusion and whether the effect was a risk factor or protective in the given model for each top model selected by the Bayesian model averaging model search method is shown in Figure 1. Variables are ordered on the vertical axis according to the posterior probability that their effect size was non-zero. These posterior probabilities, along with posterior mean odds ratios and their approximate 95% confidence intervals, conditional on those effects being included in the model, are included in Table 7.

**Table 7:** Summary statistics from Bayesian model averaging, including the posterior probability that the predictor variable effect is non-zero, the posterior mean odds ratio, and the approximate 95% posterior interval for the odds ratio, conditional on the predictor variable being included in the model.

Variable	Posterior probability the variable effect size is non-zero	Conditional posterior mean odds ratio	Conditional 95% posterior interval
Intercept		0.1	(0.0, 1.2)
Control zone	**0.55**	**10.3**	**(1.1, 100.5)**
No farm entrance gate	**0.53**	**7.0**	**(1.1, 43.7)**
Waterfowl presence	**0.40**	**6.2**	**(1.1, 39.6)**
Wild bird access to feed	0.25	5.0	(0.8, 30.8)
Flock size	0.22	5.9	(0.8, 44.3)
Off-site disposal	0.17	4.1	(0.7, 25.5)
No specific barn personnel	0.14	6.4	(0.4, 97.1)
At least some rodent problems	0.11	3.1	(0.6, 15.3)
Change of clothing not always required for workers	0.10	4.5	(0.4, 48.4)
Sharing company trucks/trailers	0.07	3.1	(0.4, 23.5)
Mowing less than 4 times/month	0.07	2.8	(0.4, 18.6)
Lower level of vehicle washing	0.07	2.7	(0.4, 20.0)

Predictor variables statistically significant at the 0.05 level are shown in bold.

Comparing the LOOCV model-based selection and ranking to BMA, the most important predictor variables were similar: control zone, no farm entrance gate, waterfowl presence, wild bird access to feed, flock size, and no specific barn personnel (probabilities of 0.55, 0.53, 0.40, 0.25, 0.22, and 0.14, respectively).

There were some differences, including off-site disposal (posterior probability of 0.17) being a moderate to low effect according to BMA, but it was not included in any of the top 15 models sorted by LOOCV, and vehicle washing, which had the lowest posterior probability of 0.07 using BMA, but was a moderate to low effect using LOOCV, being included in 4 of the top 15 models. However, many of the differences in order of importance of effects were present only for the lower and moderate sized effects, while the most important effects were the same by both modeling methods.

Odds ratios were also broadly similar between the two methods. Control zone had an OR of 10.3 (95% CI: 1.1–100.5, Table 7), which was very similar to that derived using the LOOCV-based top model. Not having a farm entrance gate had a BMA-based OR of 7.0 (95% CI: 1.1–43.7), compared to 3.8 (95% CI: 0.6–31.5) in the LOOCV-based top model, which can be partly explained by the strong confounding between control zone and the not having a farm entrance gate. Waterfowl presence had a similar estimate using BMA (OR = 6.2, 95% CI: 1.1–39.6) compared to LOOCV (OR = 5.8, 95% CI: 0.7–79.4) and not always having different personnel for different barns had a similar estimate using BMA (OR = 6.4, 95% CI: 0.4–97.1) compared to LOOCV (OR = 6.2, 95% CI: 0.3–27.5). Although not included in the top model by LOOCV, the BMA estimate for the OR for wild bird access to feed was 5.0 (95% CI: 0.8–30.8).

#### 3.2.5. Model fit diagnostics

There were no substantial indicators of poor model fit for the multiple logistic regression models, after inspection of the residual diagnostic plots for the top models.

## 4. Discussion

The wave of H5N1 clade 2.3.4.4b HPAI that began in 2021 has been unprecedented in several regions of the world (40), including the United States. As avian influenza viruses continue to circulate in wild birds, it is critical to identify measures that may help mitigate infections in domestic poultry (41, 42). The case–control study presented here investigated the risk factors associated with infection with HPAI virus between February and September 2022 on U.S. commercial table egg farms in 8 states. Although the table egg sector is a relatively small sector of the U.S. commercial poultry industry, it has been heavily affected by this outbreak. Over 80% of affected table egg producers in participating states took part in this study—a testament to the willingness of the industry to support science-based prevention efforts. It can be challenging to interpret findings from a small dataset, and so we focus on interpretation of both the outcomes of the multivariable modeling processes and the univariable analyses.

Garber et al. (12) noted that the most significant farm-level risk factor for HPAI on commercial table egg farms in 2015 was being located within an existing control zone. In the current study, this finding continues to hold true. This predictor was the closest to being significant in the exact multiple logistic regression model at the 0.05 significance level, as well as present in each of the top fifteen models produced by the LOOCV process. Farms that are located near an infected farm must be particularly diligent about biosecurity-related practices to protect flock health. Proximity to an infected farm has also been reported as a risk factor for HPAI infection in outbreaks in Europe and Japan (43, 44). Study findings confirm the need for both biosecurity and surveillance on poultry farms near an infected farm, to prevent infection and ensure rapid detection, whether the virus is likely spreading by wild birds or between farms.

Although multivariable modeling in the Garber et al. (12) study did not find an association between presence of wild birds on or around the farm and disease status, we did detect an association between sightings of wild waterfowl or shorebirds in the field closest to the farm during the reference period and farm-level disease status. Again, this predictor was present in the exact multiple logistic regression model, was in each of the top models produced by the LOOCV process, and had the third-highest posterior probability of having a non-zero effect size in BMA. Notably, this variable was a significant predictor of HPAI infection in the univariate analysis even though, as a group, all the farms that participated in the study had no other types of poultry on the farm, and none had pastured poultry or poultry with outdoor access. While this result may be due in part to recall bias by producers on case farms, producers seeking to decrease risk for HPAI may wish to work with a wildlife mitigation specialist to develop a wild bird management plan.

Included in 7 of the top models by the LOOCV process and the fourth-highest ranked predictor by BMA, any access of wild birds to feed or feed ingredients appeared to be an important predictor of HPAI farm status classification. Feed accessible to wild birds could act as a congregation point for wild birds on the farm and could increase risk of exposure to virus shed by affected wild birds. In addition, although not statistically significant, only 40% of farms that had a protocol to clean spilled feed immediately were classified as cases, while 60% of farms that had no protocol listed or a protocol to clean spilled feed less frequently were classified as cases, further supporting the need to include regular inspection of feed housing and prompt cleanup of feed spills in an overall flock management and wild bird management plan.

The presence of a farm gate was found to be protective. This predictor was present in the exact multiple logistic regression, in each of the top models produced by LOOCV, and was the predictor with the second-highest posterior probability of having a non-zero effect size by BMA. Gates were much more commonly reported on control operations than on case operations (64% vs. 22%). Having a gate may be a proxy variable for other biosecurity practices and could even be associated with a highly proactive approach to biosecurity. Gates improve control of traffic onto farms and may increase the likelihood that visitors will see posted signage and follow requested biosecurity procedures.

Flock size was non-significantly associated with increased risk in the exact multiple logistic regression (*p* = 0.23), was in 9 of the top 15 models produced by the LOOCV process, and was one of the top 5 most important predictors by BMA. This may be a finding associated with selection bias; our estimated response rate for control producers was 20%. Smaller producers may have been more likely to participate in the study.

There were two farm worker biosecurity practices that had *p* ≤ 0.20 in the univariable analysis and appeared to be low to moderately important in multivariable modeling, accounting for the other modeled effects. These were always having different personnel working in different barns (workers assigned to specific barns) and always requiring a change of clothing for workers before they enter a barn. Having workers assigned to specific barns is not a commonly reported practice, although movement of employees between barns is a known biosecurity risk (45). Having the available resources to perform biosecurity measures, such as appropriate facility design features, sufficient time, and personnel, can also affect the degree to which workers are able to carry out practices that support good biosecurity (46). Requiring a change of clothing for workers entering barns is commonly advised in biosecurity guidance, as well as in general guidance provided by United Egg Producers Animal Health and Biosecurity Committee (47).

Lack of a rodent problem was another factor more commonly reported on control farms; 28% of case farms reported having no rodent problem, whereas 54% of control farms reported no rodent problem (*p* = 0.12). The reported presence of any degree of rodent problem on-farm was a moderately important effect by both LOOCV and BMA. There is at least some evidence that rodents can transmit low-pathogenic avian influenza (48). Control of rodents is often advised to limit HPAI and other disease risks. While not meeting the criteria for confounding, mowing less frequently appeared to be related to farms having a rodent problem. That is, of farms that mowed more frequently, only 42% had a rodent problem, while of those that mowed less frequently, 71% had a rodent problem. This finding suggests that one part of an effective rodent control program could include frequent mowing of vegetation around poultry barns.

Vehicle washing was moderately important as measured by both LOOCV and BMA. This finding of moderate importance may be an artifact of the limitations of a survey-based approach; effectiveness of cleaning can be difficult to measure based on visual inspection (49). Notably, multiple vehicle wash-related variables were univariately significant (*p* ≤ 0.20), including washing of feed trucks, egg trucks, washing truck tires, and having a permanent vehicle washing station.

Historic work has noted increased risks associated with use of rendering for disposal (12, 50–53). In the current study, 33.3% of case farms reported the use of rendering for dead bird disposal, while 13.6% of control farms utilized this carcass disposal practice. Rendering vehicles may transport virus via vehicle movement from farm to farm. Additionally, depending on storage of mortalities prior to renderer pickup, there is the possibility of attracting scavengers, which can include gulls, vultures, and other wild birds. Interestingly, though not a common practice among producers, use of incineration as a disposal method was significant (*p* = 0.10) in the univariate analysis. Given the recurring finding of rendering as a risk factor in multiple outbreaks, carcass disposal practices warrant further investigation. More generally, off-site disposal may increase risk due to vehicle movement between farms: off-site disposal was reported by 50% of case farms and 27% of control farms (*p* = 0.19).

The univariate analyses of the subset of cases linked to wild bird introductions suggested that topography and proximity to bodies of water can affect risk for transmission of HPAI. Presence of a structural windbreak such as a hill was protective (*p* = 0.07), and proximity to a drainage ditch was a risk factor (*p* = 0.15), though proximity to a wastewater lagoon was not statistically significant. Seeing wild waterfowl or shorebirds in the closest field during the reference period was also associated with increased risk (*p* = 0.05). While these characteristics cannot be changed, the data suggest that risk can be mitigated by limiting areas where water can pool on and around the farm and employing wildlife mitigation strategies (54). Notably, not having a rodent problem was associated with decreased risk (*p* = 0.14), as was lack of wild bird access to feed or feed ingredients (*p* = 0.03). Further study of the effectiveness of specific mitigation practices would be valuable.

## 5. Limitations

This study has a number of general limitations. The sample size for this investigation was relatively small, with a total size of 40 observations, 18 being case farms and 22 being control farms. However, 18 of the 22 (82%) eligible commercial table egg case farms participated in the study. The estimated rate of participation was substantially lower for control farms, so practices among this group of participants may not have been representative of unaffected commercial table egg producers overall. Relatively few table egg pullet and breeder farms were affected by HPAI during the study period, so although they were included in the study, not all findings may apply to these subgroups of the table egg sector. Recall bias is another limitation. Some respondents in the study were asked to provide responses for observations and activities that had taken place months prior to the study. Recall for some questions may have been different for case farms versus control farms. Another limitation of survey-based methods is the potential for bias associated with questions that may be considered sensitive. Respondents may be more likely to provide responses considered to be aligned with best practices, rather than reflective of actual practices. While our goal was to balance the number of completed case and control questionnaires geographically, 1:1 matching of cases and controls by state was not feasible due to variation in response rates between cases and controls, as well as a lack of eligible and interested controls.

Since predictors were pre-screened prior to performing LOOCV, estimated cross-validation accuracy of the model was likely artificially inflated (32, 39). That is, the performance of the models may be lower than they are shown to be here (72.5–77.5 percent accuracy, from Table 5) if applied to a different dataset. This was acceptable because unbiased predictive ability wasn’t the end goal. Instead, the goal was to assist in selecting variables for the exact multiple logistic regression model. In addition, outbreak parameters (routes of transmission, effectiveness of control measures) can change over time or between outbreaks, therefore, the prediction error may not be directly applicable to new datasets but may serve as an adequate baseline.

Future HPAI and weather-related analysis using the 2022 outbreak data is planned across affected poultry sectors. Case–control study data will be analyzed in combination with weather conditions that occurred during the time preceding detection of HPAI infection. Weather patterns related to transmission of HPAI have been studied previously and may have had a role in the 2022 outbreak. Another direction of study underway for the turkey sector examines biosecurity investments farmers have made since 2015 and their impacts on classification of farms as cases or controls. These data will be analyzed and reported separately to identify priority areas for investment to reduce risk for HPAI. A similar study for table egg farmers may be of benefit.

## Data availability statement

The original contributions presented in the study are included in the article/Supplementary material, further inquiries can be directed to the corresponding author.

## Author contributions

AG, KM, MB, VF, KP, AB, SK, AF, and AD contributed to the conception and design of the study. MB, AG, VF, KP, MV, and RM performed the statistical analysis, with MB and MV providing focused statistical expertise. AG wrote the first draft of the manuscript. MB and KP wrote sections of the manuscript. All authors contributed to the article and approved the submitted version.

## Conflict of interest

The authors declare that the research was conducted in the absence of any commercial or financial relationships that could be construed as a potential conflict of interest.

## Publisher’s note

All claims expressed in this article are solely those of the authors and do not necessarily represent those of their affiliated organizations, or those of the publisher, the editors and the reviewers. Any product that may be evaluated in this article, or claim that may be made by its manufacturer, is not guaranteed or endorsed by the publisher.

## Footnotes

^1^ https://github.com/USDA-VS/vSNP
