# Supplementary material for: Investigation of risk factors for introduction of highly pathogenic avian influenza H5N1 virus onto table egg farms in the United States, 2022: a case–control study
Source: Front Vet Sci. 2023 Jul 25;10:1229008. doi: 10.3389/fvets.2023.1229008 (PMC10408129; doi:10.3389/fvets.2023.1229008)
Supplement: Supplementary file 1 [file Table_1.pdf]

Supplementary materials

Farm-Level Univariate Analysis

(Results do not include imputed values.)

Table S1 - Premises description

Characteristic	% Case farms (n)	% Control farms (n)	p-value (Fisher's exact)
**In existing control zone**	44.4 (8)	9.1 (2)	0.025
**Stage of production**			
Pullets OR breeders (e302*e201)	22.2 (4)	13.6 (3)	0.680
Layers (e303*e201)	83.3 (15)	90.9 (20)	0.642
**Other type(s) of poultry**			
Turkey (e306*e201)	0.0	0.0	
Broiler (e307*e201)	0.0	0.0	
**Farm certified organic (e309*e201)**	**	**	1.000
**Enrolled in NPIP (e310*e201)**	100.0 (18)	95.5 (21)	1.000
**Age**			
Multiple age (e311*e201)	88.9 (16)	72.7 (16)	0.258
Single age (e311*e201)	11.1 (2)	27.3 (6)	
**Flock size ≥500,000 (sizecat*e201)**	61.1 (11)	50.0 (11)	0.537
**Any birds with access to outdoors (e318*e201)**	0.0	0.0	
**Any pastured poultry on farm (e319*e201)**	0.0	0.0	
**Any non-poultry livestock or animals present on farm* (e320*e201)**	33.3 (6)	22.7 (5)	0.498
**Livestock, excluding poultry, on the farm, or located within 350 yards of the farm fed supplemental feed* (e321*e201)**	22.2 (4)	27.3 (6)	1.000
**Other poultry or domesticated waterfowl on farm* (e322*e201)**	0.0	0.0	
**Water source for poultry**			
Municipal (e323*e201)	16.7 (3)	13.6 (3)	1.000

Well (e324*e201)	88.2 (15)	95.0 (19)	0.584
Surface water (e.g., pond) (e325*e201)	0.0	0.0	
**Water treatments used for drinking water (e201*e327*e327a)**			1.000
Yes, continuously	55.6 (10)	40.9 (9)	
Yes, intermittently	0.0	18.2 (4)	
No	44.4 (8)	40.9 (9)	
**Evergreen or juniper windbreak present (e328*e201)**	11.1 (2)	9.1 (2)	1.000
**Deciduous tree windbreak present (e329*e201)**	55.6 (10)	36.4 (8)	0.339
**Structural windbreak present (e.g., hill, natural break) (e330*e201)**	22.2 (4)	36.4 (8)	0.491
**Water body type(s) visible or within 350 yards of farm**			
Pond (e336*e201)	22.2 (4)	40.9 (9)	0.312
Lake (e337*e201)	**	**	1.000
Stream (e338*e201)	27.8 (5)	31.8 (7)	1.000
River (e339*e201)	0.0	0.0	
Wetland or swamp or marsh (e340*e201)	16.7 (3)	22.7 (5)	0.709
Wastewater lagoon (e341*e201)	66.7 (12)	45.5 (10)	0.216
Drainage ditch (e342*e201)	55.6 (10)	36.4 (8)	0.339
**For waterbodies within 350 yards of the farm, wild waterfowl or shorebirds seen on the water* (e345cat*e201)**	50.0 (9)	35.0 (7)	0.512
**Wild waterfowl or shorebirds on closest body of water at one time* (e348cat*e201)**	64.7 (11)	50.0 (9)	0.500
**Field within 350 yards where crops or hay are harvested (e349 Y/N*e201)**	100.0 (18)	**	1.000
**Field within 33 yards of farm (median distance reported)**	61.1 (11)	40.9 (9)	0.341
**Crop grown in field within 350 yards* (e351cat*e201)**			0.098

Corn	11.1 (2)*	23.8 (5)*	
Soybeans	16.7 (3)	14.3 (3)	
Alfalfa or grass intended for livestock feed	16.7 (3)	19.0 (4)	
Cover crop – barley/wheat	27.8 (5)	0.0	
None	27.8 (5)	28.6 (6)	
Other	0.0	14.3 (3)	
**Field within 350 yards tilled last fall (e352cat*e201)**	11.1 (2)	42.9 (9)	0.038
**Closest field actively worked* (e353cat*e201)**	0.0	9.5 (2)	0.490
**Wild waterfowl or shorebirds seen in closest field* (e354cat*e201)**	44.4 (8)	9.5 (2)	0.025
**Types of waterfowl or shorebirds seen in closest field***			
Ducks (e355*e201)	50.0 (4)	**	1.000
Geese (e356*e201)	88.9 (8)	**	0.200
Shorebirds (e.g., wading birds, gulls) (e357*e201)	0.0	**	0.200

* Refers to the 14-day reference period.

**Too few to report.

Table S2 - Wild birds during the 14-day reference period*

**Characteristic**	**Level**	**% Case farms (n)**	**% Control farms (n)**	**p-value** (Fisher's exact)
**Frequency of wild birds seen on the farm but outside the barns (within 100 yards)**				
Large birds (e.g., pigeons, crows) (e359*e201)	Often	38.9 (7)	36.4 (8)	1.000
	Sometimes	44.4 (8)	50.0 (11)	
	Never	16.7 (3)	13.6 (3)	
Small birds (e.g., finches, sparrows, starlings) (e360*e201)	Often	61.1 (11)	63.6 (14)	0.377
	Sometimes	27.8 (5)	36.4 (8)	
	Never	11.1 (2)	0.0	

Waterfowl or shorebirds (e361* e201)	Often	16.7 (3)	9.1 (2)	0.822
	Sometimes	33.3 (6)	31.8 (7)	
	Never	50.0 (9)	59.1 (13)	
**Frequency of wild birds seen in the barns**				
Large birds (e.g., pigeons, crows) (e363*e201)	Often	0.0	0.0	
	Sometimes	0.0	0.0	
	Never	100.0 (18)	100.0 (22)	
Small birds (e.g., finches, sparrows, starlings) (e364*e201)	Often	**	**	1.000
	Sometimes	**	**	
	Never	94.4 (17)	90.9 (20)	
**Dead wild birds seen:**				
Large birds (e.g., pigeons, crows) (e366*e201)	Inside the barns (e366*e201)	0.0	0.0	
	Outside the barns (e369*e201)	**	**	1.000
Small birds (e.g., finches, sparrows, starlings) (e367*e201)	Inside the barns (e367*e201)	0.0	0.0	
	Outside the barns (e370*e201)	0.0	18.2 (4)	0.114

* All responses refer to the 14-day reference period.

**Too few to report.

Table S3 - Farm biosecurity

**Characteristic**	**Level**	**% Case farms (n)**	**% Control farms (n)**	**p-value** (Fisher's exact)
**House with people living in it on the property (e401*e201)**		61.1 (11)	77.3 (17)	0.315
If yes, common drive entrance to farm and residence (e402*e201)		81.8 (9)	58.8 (10)	0.250

**Road surface for vehicles coming onto operation (e404*e201)**				1.000
Hard top/asphalt		50.0 (9)	50.0 (11)	
Gravel/Dirt		50.0 (9)	50.0 (11)	
**Vehicles**				
Garbage/dumpster (e405*e201)	Come to perimeter only	33.3 (6)	13.6 (3)	0.306
	Enter farm but not near barns	50.0 (9)	50.0 (11)	
	Come near barns	16.7 (3)	27.7 (6)	
	NA	0.0	9.1 (2)	
Propane delivery (e406*e201)	Come to perimeter only/enter farm but not near barns	38.9 (7)	31.8 (7)	0.323
	Come near barns	22.2 (4)	45.5 (10)	
	NA	38.9 (7)	22.7 (5)	
Feed delivery (e407cat*e201)	Come to farm	83.3 (15)	86.4 (19)	1.000
Feed ingredient delivery (e408*e201)	Come to farm	33.3 (6)	36.4 (8)	1.000
Renderer (e409*e201)	Come to perimeter only	**	**	0.719
	Enter farm but not near barns	**	**	
	Come near barns	**	**	
	NA	88.9 (16)	95.5 (21)	
Company personnel (e.g., processing plant and barn workers, service person, veterinarian) (e410*e201)	Come to perimeter only	22.2 (4)	14.3 (3)	0.641
	Enter farm but not near barns	16.7 (3)	28.6 (6)	
	Come near barns	55.6 (10)	57.1 (12)	
Egg trucks moving eggs off the farm (e.g., to processing, to breaking, to the consumer market) (e411cat*e201)	Come to farm	83.3 (15)	20 (95.2)	0.318

Egg truck moving eggs to the farm (e.g., sideloading) (e412cat*e201)	Come to farm	22.2 (4)	23.8 (5)	1.000
Other business visitors (e.g., meter reader, repairman) (e413cat*e201)	Come to farm	61.1 (11)	77.3 (17)	0.315
**Farm entrance gated (e417*e201)**		22.2 (4)	63.6 (14)	0.012
If yes, gate secured/locked (e418*e201)	Always	75.0 (3)	50.0 (7)	0.764
	After hours only	**	21.4 (3)	
	Never	**	28.6 (4)	
**Farm area perimeter surrounded by security fence (e419*e201)**		17.7 (3)	18.2 (4)	1.00
**Frequency vegetation mowed/ bush hogged (e420cat*e201)**				0.200
Less than 4 times a month		64.7 (11)	40.9 (9)	
4 or more times a month		35.3 (6)	59.1 (13)	
**Wash station or spray area used for vehicles* (e421*e201)**		72.2 (13)	81.8 (18)	0.705
If yes, vehicle wash station or spray area:*	Located on the farm (e422*e201)	92.3 (12)	100.0 (18)	0.419
	Tires washed (e423*e201)	84.6 (11)	100.0 (18)	0.168
	Vehicle exterior washed (e424*e201)	23.1 (3)	27.8 (5)	1.000
	Vehicle interior cleaned (e425*e201)	15.4 (2)	16.7 (3)	1.000
	Worker vehicles washed (e426*e201)	61.5 (8)	77.8 (14)	0.433
	Feed trucks washed (e427*e201)	69.2 (9)	100.0 (18)	0.023
	Egg trucks washed (e428*e201)	69.2 (9)	94.1 (16)	0.138
	All vehicles washed (e429oth*e201)	28.6 (2)	46.7 (7)	0.648

	Recently put into use as a response to heightened biosecurity concerns (e431*e201)	76.9 (10)	50.0 (9)	0.158
	A permanent station (such as in use prior to HPAI incident) (e431*e201)	23.1 (3)	50.0 (9)	0.158
Distance from vehicle wash station to nearest barn (meters)* (e432*e201)	≤50 meters	61.5 (8)	38.9 (7)	0.285
	More than 50 meters	38.5 (5)	61.1 (11)	
**Workers park in restricted area away from barns* (e433*e201)**	Always	61.1 (11)	68.2 (15)	0.643
	Sometimes	27.8 (5)	13.6 (3)	
	Never	11.1 (2)	18.2 (4)	
**Visitors always park in restricted area away from barns* (e434*e201)**		77.8 (14)	72.7 (16)	1.000
**Rat and mouse bait stations used* (e435*e201)**		100.0 (18)	100.0 (22)	
**Beetle control used* (e437*e201)**		**	18.2 (4)	0.363
**Any problem with rodents* (e445cat*e201)**	Any problem (high, moderate, low)	72.2 (13)	45.5 (10)	0.116
**Rodent index monitored (e446*e201)**		**	100.0 (22)	0.450
If yes, range of rodent index* (e447*e201)	Low (0 to 10 mice)	75.0 (12)	90.5 (19)	0.371
	Medium/High (11 or more mice)	25.0 (4)	9.5 (2)	
**Wild mammals or evidence seen in or around barns* (e448*e201)**		16.7 (3)	13.6 (3)	1.000
**Wild birds able to access feed/feed ingredients* (e449*e201)**	Bird access	50.0 (9)	27.3 (6)	0.194
**Wild animals (e.g., raccoons, opossums, coyotes, foxes) able to access feed/feed ingredients* (e450*e201)**	Wild animal access	33.3 (6)	9.1 (2)	0.112

**Rodents able to access feed/feed ingredients* (e451*e201)**	Always/Most of the time	22.2 (4)	13.6 (3)	0.837
	Sometimes	38.9 (7)	40.9 (9)	
	Never	38.9 (7)	45.5 (10)	
**Protocol for feed spills (describe) (e453 categories * e201)**	Immediately	64.7 (11)	81.0 (17)	0.293
	Daily or weekly, missing	35.3 (6)	19.0 (4)	
**Wildlife management plan in place (e452*e201)**	Yes	55.6 (10)	40.9 (9)	0.690
	No	33.3 (6)	40.9 (9)	
	Don't know	11.1 (2)	18.2 (4)	
**Form of feed fed**	Mash (e454*e201)	94.1 (16)	95.5 (21)	1.000
	Pellets (e455 * e201)	0.0	0.0	
**Feed treated with**	Formaldehyde (e457 *e201)	**	**	0.579
	Antimicrobial (e.g., ionophores) (e458 *e201)	**	**	1.000
**Feed heat treated**	(e460*e201)	0.0	0.0	

* Responses refer to the 14-day reference period.

**Too few to report.

Table S4 - Farm help/workers

Characteristic	Level	% Case farms (n)	% Control farms (n)	p-value (Fisher's exact)
**Use occasional or emergency workers to fill in* (e602*e201)**		11.8 (2)	9.1 (2)	1.000
**Measures required for workers entering the barns:***				
Established clean/dirty line (e603*e201)	Always	83.3 (15)	90.9 (20)	0.642
	Most of the time/sometimes			
	Never	0.0	0.0	

Shower (e604*e201)	Always/most of the time	33.3 (6)	22.7 (5)	0.554
	Sometimes	0.0	9.1 (2)	
	Never	66.7 (12)	68.2 (15)	
Wash hands or use hand sanitizer before entering barn (e605*e201)	Always	72.2 (13)	54.6 (12)	0.332
Different personnel for different barns (e606 *e201)	Always/most of the time	38.9 (7)	50.0 (11)	0.764
	Sometimes	16.7 (3)	13.6 (3)	
	Never	44.4 (8)	36.4 (8)	
Wear disposable coveralls (e607*e201)	Always/most of the time	16.7 (3)	9.1 (2)	0.501
	Sometimes	11.1 (2)	27.3 (6)	
	Never	72.2 (13)	63.6 (14)	
Change of clothing (washable) (e608*e201)	Always	66.7 (12)	90.9 (20)	0.124
	Most of the time	**	0.0	
	Sometimes	**	0.0	
	Never	16.7 (3)	9.1 (2)	
Change of shoes or use of shoe covers (e609cat*e201)	Always	100.0 (18)	95.5 (21)	1.000
Foot bath (liquid) (e610*e201)	Always	27.8 (5)	31.8 (7)	0.921
	Most of the time	0.0	**	
	Sometimes	0.0	**	
	Never	72.2 (13)	59.1 (13)	
Foot bath (dry) (e611cat*e201)		77.8 (14)	68.2 (15)	0.724
Scrub footwear (bucket and brush) (e612cat*e201)	Always/most of the time	16.7 (3)	13.6 (3)	0.102
	Sometimes	0.0	22.7 (5)	
	Never	83.3 (15)	63.6 (14)	
**Policy in place to prevent workers from working on another poultry farm (e613*e201)**		100.0 (18)	95.5 (21)	1.000

**Workers or household members employed by other poultry operations, rendering plants, processing plants (e614*e201)**		**	**	0.450
**Employees own their own poultry, including backyard flocks (e615*e201)**	Yes	**	0.0	0.450
	No	94.4 (17)	100.0 (22)	
	Don't know	**	0.0	
**Employees required to stay off farm after exposure to other poultry (e616*e201)**		94.4 (17)	86.4 (19)	0.613

* Responses refer to the 14-day reference period.

**Too few to report.

Table S5 - Farm visitors during the 14-day reference period*

**Visitor type**	**Visited the farm/entered barn during the 14-day reference period**	**% Case farms (n)**	**% Control farms(n)**	**p-value** (Fisher's exact)
Federal/State veterinary or animal health worker	Visited farm (e702*e201)	22.2 (4)	9.1 (2)	0.381
	Entered barn (e738*e201)	75.0 (3)	100.0 (2)	1.000
Extension agent or university veterinarian	Visited farm (e703*e201)	0.0	**	1.000
	Entered barn (e739*e201)	0.0	**	
Private or company veterinarian	Visited farm (e704*e201)	29.4 (5)	14.3 (3)	0.426
	Entered barn (e740*e201)	0.0	100.0 (3)	0.018

Company service person	Visited farm (e705*e201)	55.6 (10)	54.6 (12)	1.000
	Entered barn (e741*e201)	75.0 (6)	91.7 (11)	0.537
Nutritionist or feed company consultant	Visited farm (e706*e201)	0.0	13.6 (3)	0.243
	Entered barn (e742*e201)	0.0	**	
Feed ingredient delivery person	Visited farm (e707*e201)	22.2 (4)	22.7 (5)	1.000
	Entered barn (e743*e201)	0.0	0.0	
Pullet delivery	Visited farm (e708*e201)	11.1 (2)	9.1 (2)	1.000
	Entered barn (e744*e201)	100.0 (2)	100.0 (2)	
Vaccination crew	Visited farm (e709*e201)	**	**	1.000
	Entered barn (e745*e201)	**	**	
Catch crew	Visited farm (e710*e201)	**	18.2 (4)	0.356
	Entered barn (e746*e201)	**	100.0 (4)	
Feed delivery personnel	Visited farm (e711*e201)	83.3 (15)	81.8 (18)	1.000
	Entered barn (e747*e201)	15.4 (2)	0.0	0.168
Egg truck personnel	Visited farm (e712*e201)	72.2 (13)	86.4 (19)	0.430
	Entered barn (e748*e201)	0.0	11.1 (2)	0.512
Bedding/new litter services (delivery, pick-up)	Visited farm (e713*e201)	0.0	0.0	
	Entered barn (e749*e201)	0.0	0.0	

Customer (private individual)	Visited farm (e714*e201)	0.0	0.0	
	Entered barn (e750*e201)	0.0	0.0	
Wholesaler, buyer, or dealer	Visited farm (e715*e201)	0.0	0.0	
	Entered barn (e751*e201)	0.0	0.0	
Renderer	Visited farm (e716*e201)	**	**	1.000
	Entered barn (e752*e201)	0.0	0.0	
Dead bird pickup other than by renderer	Visited farm (e717*e201)	16.7 (3)	18.2 (4)	1.000
	Entered barn (e753*e201)	0.0	0.0	
Occasional worker (e.g., family member, part-time help over holiday)	Visited farm (e718*e201)	**	**	1.000
	Entered barn (e754*e201)	**	**	1.000
Construction workers, repair or maintenance personnel	Visited farm (e719*e201)	25.0 (4)	14.3 (3)	0.437
	Entered barn (e755*e201)	**	**	0.429
Other business visitors (e.g., other producers, meter readers, package delivery (UPS), propane, etc.)	Visited farm (e720*e201)	52.9 (9)	61.9 (13)	0.743
	Entered barn (e756*e201)	0.0	0.0	
Other nonbusiness visitors (including neighbors, family members, friends, school field trips)	Visited farm (e721*e201)	**	**	1.000
	Entered barn (e757*e201)	0.0	0.0	

* All responses refer to the 14-day reference period.

**Too few to report.

Table S6 - Farm visitors

**Characteristic**	**Level**	**% Case farms (n)**	**% Control farms (n)**	**p-value** (Fisher's exact)
**Visitor log used (e701*e201)**		100.0 (17)	100.0 (22)	
**Requirements of visitors who entered the barn during the 14-day reference period:**				
Change of outer clothing/farm specific clothing (e758*e201)	Yes, verified at farm	91.7 (11)	71.4 (15)	0.525
	Yes, visitor responsibility	*	9.5 (2)	
	No	*	19.1 (4)	
Foot covers or change of footwear (e759*e201)	Yes, verified at farm	91.7 (11)	75.0 (15)	0.505
	Yes, visitor responsibility	*	10.0 (2)	
	No	*	15.0 (3)	
Mask (e760*e201)	Yes	25.0 (3)	36.8 (7)	0.697
	No	75.0 (9)	63.2 (12)	
Hand sanitizing, handwashing, or gloves (e761*e201)	Yes, verified at farm	91.7 (11)	84.2 (16)	1.000
	Yes, visitor responsibility	*	*	
	No	*	*	
Not visit multiple farms in same day (e762*e201)	Yes, verified at farm	83.3 (10)	84.2 (16)	0.555
	Yes, visitor responsibility	*	0.0	
	No	*	15.8 (3)	

*Too few to report.

Table S7 - Farm vehicles and equipment

**Factor**	**Level**	**% Case farms (n)**	**% Control farms (n)**	**p-value** (Fisher's exact)
**Vehicles shared during the 14-day reference period:**				
Company trucks/trailers (e.g., pickup truck, trailer with supplies, supervisor truck, or similar) (e201* e801*e801a)	Not shared or shared and always disinfected	66.7 (12)	90.9 (20)	0.110

Feed trucks (e201* e802*e802a)	Not shared or shared and always disinfected	83.3 (15)	81.8 (18)	1.000
Feed ingredient truck (e201*e803*e803a)	Not shared or shared and always disinfected	100.0 (18)	86.4 (19)	0.239
Pullet delivery vehicles (e.g., placing pullets) (e201*e804*e804a)	Shared, disinfected	*	*	1.000
	Shared, sometimes disinfected	*	*	
	Shared, not disinfected	*	*	
	Not shared	94.4 (17)	90.9 (20)	
Bird removal vehicles (e201*e805*e805a)	Shared, disinfected	*	*	0.490
	Shared, sometimes disinfected	*	*	
	Shared, not disinfected	*	*	
	Not shared	100.0 (18)	85.7 (21)	
Egg delivery vehicles (e201*e806*e806a)	Shared, disinfected	*	*	0.883
	Shared, sometimes disinfected	*	*	
	Shared, not disinfected	*	*	
	Not shared	72.2 (13)	61.9 (13)	
Egg removal vehicles (e201*e807*e807a)	Not shared or shared and always disinfected	77.8 (14)	81.8 (18)	1.000
Manure/litter hauling (e201*e808*e808a)	Shared, disinfected	*	*	1.000
	Shared, sometimes disinfected	*	*	
	Shared, not disinfected	*	*	
	Not shared	72.2 (13)	72.7 (16)	
ATV/4-wheeler (e201*e809*e809a)	Shared, disinfected	0	0	-
	Shared, sometimes disinfected	0	0	
	Shared, not disinfected	0	0	
	Not shared	100.0 (18)	100.0 (22)	
**Equipment shared during the 14-day reference period:**				
Gates/panels (e201*e811*e811a)	Shared, disinfected	0	0	-
	Shared, sometimes disinfected	0	0	

	Shared, not disinfected	0	0	
	Not shared	100.0 (18)	100.0 (22)	
Lawn mowers (e201*e812*e812a)	Not shared or shared and always disinfected	100.0 (18)	100.0 (22)	-
Live haul loaders (e201*e813*e813a)	Not shared or shared and always disinfected	100.0 (17)	100.0 (22)	-
Egg racks or pallets (e201*e814*e814a)	Not shared or shared and always disinfected	100.0 (18)	86.4 (19)	0.239
Egg flats (e201*e815*e815a)	Not shared or shared and always disinfected	100.0 (17)	90.9 (20)	0.495
Pressure sprayers/washers/foamers (e201*e816*e816a)	Shared, disinfected	0	0	-
	Shared, sometimes disinfected	0	0	
	Shared, not disinfected	0	0	
	Not shared	100.0 (18)	100.0 (22)	
Skid-steer loaders (e201*e817*e817a)	Not shared or shared and always disinfected	100.0 (18)	100.0 (22)	-
Litter/manure handling (e201*e818*e818a)	Shared, disinfected	*	*	0.806
	Shared, sometimes disinfected	*	*	
	Shared, not disinfected	*	*	
	Not shared	83.3 (15)	86.4 (19)	
Mortality bin (e201*e819*e819a)	Not shared or shared and always disinfected	100.0 (18)	100.0 (22)	-

*Too few to report.

Table S8 - Egg handling

**Characteristic**	**Level**	**% Case farms (n)**	**% Control farms (n)**	**p-value** (Fisher's exact)
Eggs marketed as shell eggs* (e901*e201)		61.1 (11)	68.2 (15)	0.744
	If yes, washed and sanitized eggs* (e902*e201)	81.8 (9)	60.0 (9)	0.395

	If yes, nest run eggs* (e903*e201)	18.2 (2)	46.7 (7)	0.217
Eggs marketed as liquid eggs (sent to further processing)* (e904*e201)		27.8 (5)	45.5 (10)	0.332
Both shell and liquid eggs marketed*		11.1 (2)	18.2 (4)	0.673
Primary location for shell egg processing (washing, grading, packing) (e905*e201)	On-farm	64.7 (11)	45.0 (9)	0.325
	Off-farm	35.3 (6)	55.0 (11)	
For on-farm processing, shell eggs from other farms processed on this farm (e.g., side-loading) (e906*e201)		**	**	1.000
For off-farm processing, poultry present on property where processing facility is located (e909*e201)		**	**	1.000

* Responses refer to the 14-day reference period.

**Too few to report.

Table S9 - Litter handling

**Characteristic**	**Level**	**% Case farms (n)**	**% Control farms (n)**	**p-value**
Litter (bedding) used on farm (e1001*e201)		11.1 (2)	22.7 (5)	0.427
Litter brought on farm* (e1002*e201)		0.0	0.0	-
Who brings litter on farm (e1003*e201)	Company provider	**	**	1.000
	Litter provider	**	**	
Litter heat treated prior to delivery (e1004*e201)		**	**	1.000
Litter stored on farm prior to use (e201*e1005*e1006)	Outside - covered	0.0	0.0	-
	Outside – not covered	0.0	0.0	
	Not stored outside	100.0 (2)	100.0 (5)	

(e201*e1007*e1008)	In a shed	**	**	0.333
Prior to use, fresh litter accessible to:	Wild birds (e1010*e201)	**	**	1.000
	Wild animals (e1011*e201)	**	**	1.000
	Domestic animals (e1012*e201)	0.0	0.0	-

* Responses refer to the 14-day reference period.

** Too few to report.

Table S10 - Manure handling

Practice	Level	% Case farms (n)	% Control farms (n)	p-value
**Manure/used litter spread on this farm or adjacent farms prior to or during reference period (e1014*e201)**		0.0	13.6 (3)	0.239
**Manure handling methods**	High rise (pit at ground level with house above) (e1016*e201)	55.6 (10)	36.4 (8)	0.339
	Deep pit (below ground) (e1017*e201)	*	*	1.000
	Shallow pit (ground level) (e1018*e201)	*	*	0.450
	Raised slats over floor (no manure belt) (e1019*e201)	*	*	1.000
	Flush system to a lagoon or slurry pit (e1020*e201)	0.0	0.0	-
	If yes, lagoon water used to flush barns (e1021*e201)	-	-	-
	Manure belt (e1022*e201)	72.2 (13)	68.2 (15)	1.000
	Scraper system (not flush or pit) (e1023*e201)	22.2 (4)	18.2 (4)	1.000
	Drop board (e1024*e201)	*	*	0.450

**Manure stored on farm (not including high-rise pits)**	(e1026*e201)	66.7 (12)	76.2 (16)	0.723
If yes, manure stored in:	An enclosed building (e1027*e201)	50.0 (6)	62.5 (10)	0.702
	In an open structure (e.g., 3-sided building) (e1028*e201)	54.5 (6)	50.0 (8)	1.000
	In a lagoon (e1029*e201)	*	*	0.407
	Outside other than lagoon (e1030*e201)	0.0	0.0	-
Manure disposal during the 14-day reference period	Composted on-farm	*	25.0 (5)	0.196
	Applied to land on this farm (e1035*e201)	0.0	15.0 (3)	0.234
	Taken off-site (e1037*e201)	35.3 (6)	40.0 (8)	1.000

*Too few to report.

Table S11 - Dead bird disposal

**Characteristic**	**Level**	**% Case farms (n)**	**% Control farms (n)**	**p-value**
**Methods(s) of daily mortality:**				
Composting (e1102*e1102a*e201)	On-site	43.8 (7)	47.6 (10)	1.000
	Off-site	0.0	0.0	
	No	56.2 (9)	52.4 (11)	
Burial (e1103*e1103a*e201)	On-site	11.1 (2)	0.0	0.196
	Off-site	0.0	0.0	
	No	88.9 (16)	100.0 (22)	
Incineration (e1104*e201)		**	27.3 (6)	0.105
Rendering (e1105*e201)		33.3 (6)	13.6 (3)	0.253
Landfill (e1106*e201)		**	**	1.000
**If composting or burial is a method of daily mortality disposal, carcasses are:**	Covered daily or every 2 or more days with soil	**	27.3 (3)	0.587
	Never covered with soil	90.0 (9)	72.7 (8)	
	Covered daily with manure	80.0 (8)	63.6 (7)	0.522

	Covered every 2 or more days with manure	0.0	18.2 (2)	
	Never covered with manure	20.0 (2)	18.2 (2)	
**If rendering is a method of daily mortality disposal,**	Carcass bin kept covered (e201*e1105*e1110)	6 (100.0)	2 (66.7)	0.333
	Producer/worker takes carcasses to renderer (e201*e1105*e1111)	3 (60.0)	**	1.000
	Carcasses picked up from farm by renderer (e201*e1105*e1111)	2 (40.0)	**	
**Wild birds seen around dead bird collection area* (e1114*e201)**		3 (16.7)	6 (27.3)	0.476
**Wild mammals seen around dead bird collection area* (e1115*e201)**		2 (11.1)	2 (9.1)	1.000

* Responses refer to observations during the 14-day reference period.

**Too few to report.
